# Salivary Cobalt, Nickel, Chromium and Titanium After Fixed Orthodontic Appliance Removal: A 3-Month Pilot Longitudinal Study

**DOI:** 10.3390/dj14080481

**Published:** 2026-08-05

**Authors:** Brenda Jazmín Valdez-Vargas, Edith Lara-Carrillo, Efraín Rubio-Rosas, Wael Hegazy-Hassan, Víctor Hugo Toral-Rizo, Elias Nahum Salmerón-Valdés, Isabel de Monserrat Osorio-Bernal, Estefania Sicco, Ronell Bologna-Molina, Gloria Elena Guzmán-Celaya, Violeta Evelyn Flores-Solano, Ana Miriam Santillán-Reyes

**Affiliations:** 1 Health Sciences Program, Research Center and Advanced Studies in Dentistry “Dr. Keisaburo Miyata”, Faculty of Dentistry, Autonomous University of the State of Mexico, Toluca 50130, Mexico; bvaldezvargas@yahoo.com.mx; 2 Department of Orthodontics, Research Center and Advanced Studies in Dentistry “Dr. Keisaburo Miyata”, Faculty of Dentistry, Autonomous University of the State of Mexico, Toluca 50130, Mexico; 3 Knowledge Transfer and Innovation Direction, Benemerite Autonomous University of Puebla, Heroica Puebla de Zaragoza 72592, Mexico; efrain.rubio@correo.buap.mx; 4 Faculty of Dentistry, Autonomous University of the State of Mexico, Toluca 50130, Mexico; whhassanm001@profesor.uaemex.mx (W.H.-H.); ensalmeronv@uaemex.mx (E.N.S.-V.); isabelosoriob@gmail.com (I.d.M.O.-B.); violeta303@hotmail.com (V.E.F.-S.); amsantillanr@uaemex.mx (A.M.S.-R.); 5 Department of Oral Medicine and Bucal Pathology, Faculty of Dentistry, Autonomous University of the State of Mexico, Toluca 50130, Mexico; 6 Department of Diagnosis in Pathology and Oral Medicine, Faculty of Dentistry, University of the Republic of Uruguay, Montevideo 11600, Uruguay; estefania.sicco@pedeciba.edu.uy (E.S.); ronellbologna@odon.edu.uy (R.B.-M.); 7 Research Department, School of Dentistry, Juarez University of Durango State, Durango 34070, Mexico; 8 Faculty of Dentistry, Autonomous University of Sinaloa, Culiacán 80010, Mexico; gloriaguzman@uas.edu.mx

**Keywords:** optical emission spectrometry, metal ions, orthodontic appliances, saliva

## Abstract

**Objective:** Our objective was to determine the salivary levels of cobalt, nickel, chromium, and titanium ions at the end of fixed orthodontic treatment and during the post-removal period (1 and 3 months after removal of the fixed appliance). **Methods:** This pilot longitudinal study included fifteen individuals (10 females and 5 males). Five milliliters of saliva was collected at three time points: T1 (before withdrawal of orthodontic appliances), T2 (one month post-withdrawal), and T3 (three months post-withdrawal). The concentrations of nickel (Ni), titanium (Ti), chromium (Cr), and cobalt (Co) in the samples were analyzed using Inductively Coupled Plasma Optical Emission Spectroscopy (ICP-OES). All samples were analyzed in triplicate. The data were analyzed by repeated-measures ANOVA, and differences were considered significant if the *p*-value was ≤0.05. **Results:** The concentrations of Co, Cr, and Ti at the three time points were low and decreased after the removal of the orthodontic appliance, while Ni showed an increase at T3. Co and Ni showed significant differences between T3 and T1 (*p* = 0.016 and *p* = 0.029, respectively). There was also significantly increased Ni ion release at T3 compared to T2 (*p* = 0.010). The Cr and Ti levels at all three time points were significantly different (Cr: *p* = 0.001; Ti: *p* = 0.001 and *p* = 0.005). **Conclusions:** The levels of cobalt, chromium, and titanium in saliva decreased after removal of the orthodontic appliance, whereas nickel increased at T3. However, the levels were below the mean dietary intake levels; therefore, these concentrations can be considered non-toxic. This study has limitations that should be considered when interpreting the results.

## 1. Introduction

The most commonly used materials for fixed orthodontic appliances—namely, brackets, bands, archwires, and ligatures—are stainless steel (SS) alloys, with 17% to 22% chromium and 8% to 12% nickel, and nickel–titanium (NiTi) alloys, with 55% Ni and 45% Ti. Nickel is added to austenitic stainless steel to improve corrosion resistance and reduce ductility, while chromium is added to facilitate the formation of a passive anti-corrosion film [1,2]. Archwires made from other materials, such as cobalt–chromium alloys or ß-titanium alloys (ß-Ti), are less frequently used. Clinical and in vivo studies have demonstrated that different nickel-titanium wire types release varying amounts of nickel after oral exposure, underscoring the need for clinical biomonitoring across different treatment stages [1,2,3,4].

The most important factor when selecting orthodontic appliances is the biocompatibility of the materials [4,5,6,7]. Biocompatibility is the ability of a biomaterial to perform a desired function in the human body without causing undesirable local or systemic effects [6,8]. For example, the release of metal ions can lead to undesirable concentrations in human tissues and elicit a hypersensitivity response in some patients [5].

Fixed orthodontic appliances are exposed to potentially corrosive environments for periods that may extend to two years or longer; this exposure to degradation processes is a topic of interest when studying their potential side effects [6].

Continuous changes in temperature, pH, salivary conditions, microbiological activity, oral flora, and the presence/absence of food and its physical and chemical characteristics, as well as the presence of plaque, can destroy the protective layer on fixed orthodontic appliances, promoting corrosion of the dental materials [5,7,9,10,11,12,13,14]. Metal alloys in the oral cavity are also subjected to mechanical stress from masticatory forces and from the archwire sliding along the bracket slot during orthodontic treatment. This, along with saliva, can accelerate and intensify corrosion of the metallic surface, which may result in elevated concentrations of metal ions in the oral cavity [5,6,7,9,15].

Cobalt, nickel, chromium, and titanium ions released from fixed orthodontic appliances could act as allergens or cause serious biological effects. Additionally, they can be cytotoxic, mutagenic, and/or carcinogenic at low concentrations [1,2,5,10]. Some pathologies, such as mucosal erythema, allergic contact dermatitis, contact stomatitis, periodontitis, gingival hyperplasia, glossitis, gingivitis, and erythema multiforme during orthodontic treatment, may be caused by the release of metal ions from appliances [1,2,3,4,5,10,11,12,13]. While studies have examined the release of metal ions from fixed orthodontic appliances at the beginning and during treatment, no studies have examined their release after treatment, that is, at the time of maximum exposure.

This study aimed to determine the salivary levels of cobalt, nickel, chromium, and titanium ions at the end of fixed orthodontic treatment and during the post-removal period (1 and 3 months after removal of the fixed appliance).

## 2. Materials and Methods

### 2.1. Selection and Description of Participants

This pilot longitudinal study included 15 individuals (10 women and 5 men; mean age: 23.4 ± 5.3 years) (Table 1). Each participant served as their own control over three months following the completion of orthodontic treatment at the Orthodontic Postgraduate Clinic of the Research Center and Advanced Studies in Dentistry (CIEAO) “Dr. Keisaburo Miyata” at the Autonomous University of the State of Mexico, as well as at private clinics in Toluca City, Mexico. These participants met the following inclusion criteria: willingness to participate, permanent dentition (excluding third molars), absence of systemic diseases, no history of allergic reactions, no history of medication intake, no history of tobacco or alcohol consumption, and absence of caries. All participants used the same type of bracket (Roth Technique, Nu-Edge Cobalt Chromium Brackets, TP Orthodontics, Inc., La Porte, IN, USA) and 0.012”, 0.014”, 0.016”, or 0.018” Reflex Nickel Titanium wire (TP Orthodontics, Inc., La Porte, IN, USA) in the first stage of their orthodontic treatment. For the second and third stages, 0.016” × 0.022” and 0.017” × 0.022” stainless steel alloys (TP Orthodontics, Inc., La Porte, IN, USA) were used to complete treatment. The average duration of orthodontic treatment was 2 to 3 years depending on the case’s complexity. A 30-month cutoff was established to distinguish between standard treatment durations and those exceeding the average, which is useful for assessing prolonged exposure to factors such as metal ion release from orthodontic appliances. However, in the present study, no patients were treated for more than 30 months (Table 1). Participants who did not require any auxiliary metal appliance or attachment as part of their orthodontic treatment were included. The patients, their parents, or legal guardians provided signed informed consent. The convenience sample size was chosen because this study was designed as an exploratory study given the lack of prior data on the later stages of orthodontic treatment, and the sample was selected using strict criteria. Furthermore, the cohort design with longitudinal follow-up allowed each patient to serve as their own control, increasing the statistical efficiency. Studies on early-stage orthodontic treatment with similar sample sizes have been published. This study was approved by the Ethics Committee of CIEAO (registration number CEICIEAO-2024-002) on 17 May 2024.

### 2.2. Saliva Sample Preparation and Collection

Participants were recruited between August and December 2024. Unstimulated saliva samples were collected between 9:00 and 11:00 a.m. for all participants, following a standardized protocol to minimize circadian variation in salivary flow and composition. Each patient was instructed not to eat or drink before sample collection. Unstimulated saliva was collected after the participant rinsed their mouth with drinking water and waited 3 min. Five milliliters of unstimulated saliva were collected in polyethylene bottles upon the patient’s arrival and immediately stored at −20 °C until processing to prevent bacterial growth. This procedure was repeated three times: T1 (removal of the fixed orthodontic appliance), T2 (one month after removal), and T3 (three months after removal). Baseline measurements before orthodontic treatment were not obtained.

The study timeline was designed to assess short-term changes in metal-ion levels in unstimulated saliva following the removal of fixed orthodontic appliances. Measurements taken at appliance removal (T1) reflect the intraoral environment after long-term exposure to orthodontic alloys and the potential immediate release of ions during mechanical removal and debonding. The one-month check (T2) was included to evaluate the early post-treatment phase, allowing enough time for initial salivary clearance and stabilization of the oral environment after removal of the metallic source. Lastly, the three-month point (T3) was chosen as a relevant interval to assess whether salivary metal ion levels return to baseline environmental levels once the appliances are removed. This follow-up also allows measurement of any remaining ions after stopping long-term intraoral exposure to orthodontic metals, providing insight into short-term changes in metal ion levels in saliva after treatment.

Removal of fixed orthodontic appliances was standardized across all participants and performed by the same clinician. Brackets and residual adhesive were removed using only bracket removers and resin removal pliers. No rotary instruments, carbide burs, polishing systems, or metal finishing devices were used during removal. Saliva samples were collected according to the predefined study schedule after appliance removal (T1), at one month (T2), and at three months (T3). Procedural control samples and reagents were included during sample preparation and ICP-OES analysis to monitor potential contamination throughout the analytical process.

High-density polyethylene (HDPE) flasks were used to collect unstimulated saliva samples. These flasks were first washed with an alkali-extract neutral soap (Merck, Darmstadt, Germany) and then soaked in 5% (*v*/*v*) hyperpure nitric acid for 24 h to remove any heavy-metal contaminants. Finally, they were rinsed with double-distilled water and dried at room temperature.

### 2.3. Salivary Analysis

**Figure 1:**
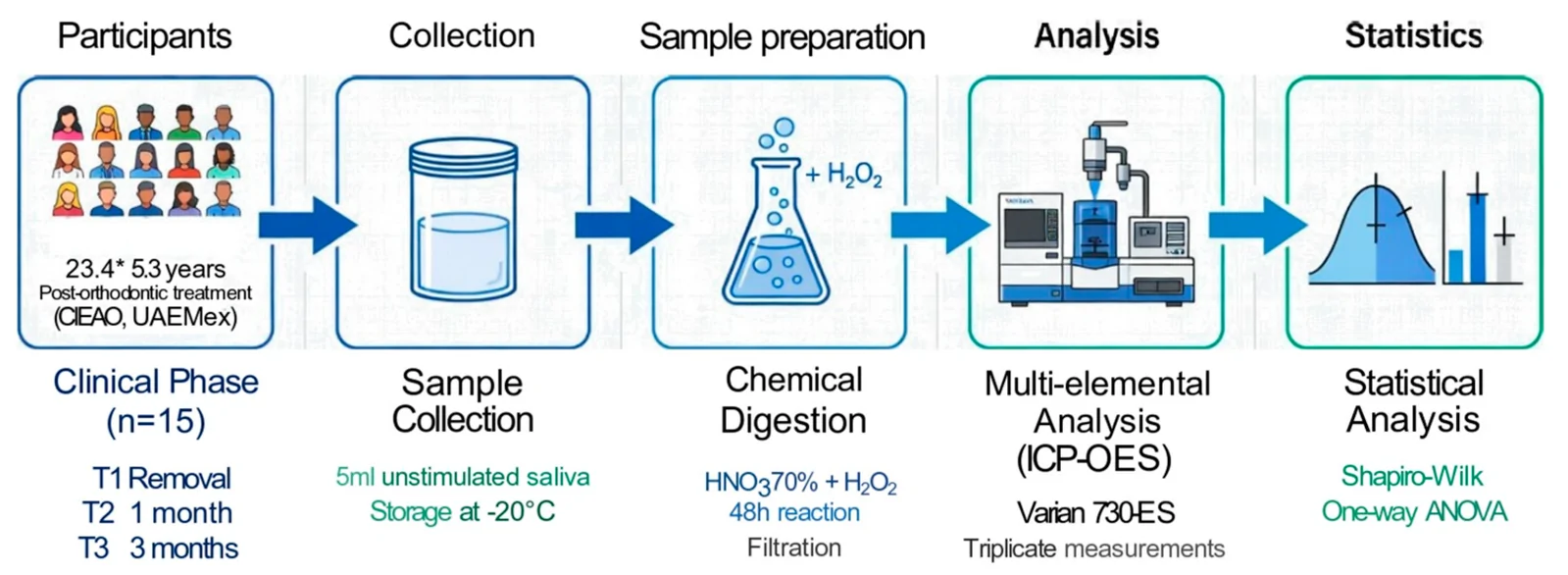
Schematic representation of the clinical and analytical workflow of the evaluation of metal ion release in saliva following orthodontic treatment.

To the unstimulated saliva collection flasks, 2 mL of H_2_O_2_ and 5 mL of 70% HNO_3_ were added, gently mixed, and left to rest for 48 h to complete digestion. Subsequently, the samples were diluted with 50 mL of H_2_O and then left to rest for 24 h. The samples were then filtered to remove heavy particles and analyzed for multiple elements using Inductively Coupled Plasma Optical Emission Spectroscopy (ICP-OES) (VARIAN 730-ES, Varian Inc., Palo Alto, CA, USA). The concentrations of the analyzed metal ions were calculated in micrograms per milliliter (μg/mL) (Figure 1).

### 2.4. Quality Control of Triplicate ICP-OES Measurements

All saliva samples were analyzed in triplicate by ICP-OES. For each sample, element, and time point, the mean concentration, standard deviation, and coefficient of variation (CV%) were calculated. Replicate precision was considered acceptable when CV ≤ 10% for concentrations above the LOQ. When CV exceeded this threshold, the raw emission signals, blank response, calibration verification, background correction, and potential spectral interferences were reviewed. Samples were re-analyzed if sufficient aliquot remained. Replicate values were excluded only when an identifiable analytical error was documented. For concentrations near the LOQ, higher CV values were interpreted cautiously, given the expected increase in relative variability at trace levels. The final reported value was the mean of analytically valid replicate measurements.

### 2.5. Analytical Validation of the ICP-OES Method

The ICP-OES method for quantifying Co, Ni, Cr, and Ti ions in saliva collected at appliance removal (T1), 1 month (T2), and 3 months (T3) after orthodontic treatment was analytically validated before sample analysis. Calibration curves were linear across the working range (0–10 mg/L), with excellent linearity for all analytes (Co: r = 0.999284–0.999409; Cr: r = 0.999324–0.999480; Ni: r = 0.999153–0.999318).

External calibration was performed using multi-element standards spanning 0 to 10 mg·L^−1^, yielding linear calibration curves with correlation coefficients (R) > 0.999 for all analytes. The analytical wavelengths were 228.615 nm (Co), 216.555 nm (Ni), 267.716 nm (Cr), and 334.941 nm (Ti).

The limits of detection (LOD) and quantification (LOQ) were calculated from seven independent measurements of blank samples (n = 7) using the 3σ and 10σ criteria, respectively. The LOD values were 10.30, 11.85, 15.74, and 8.67 µg·L^−1^ for Co, Ni, Cr, and Ti, respectively, while the LOQ values were 34.35, 39.49, 53.22, and 28.89 µg·L^−1^. Reagent and procedure blanks were included throughout the analytical sequence for quality control. The method demonstrated intra-assay coefficients of variation of less than 3% and inter-assay coefficients of variation of approximately 2%, confirming the accuracy, precision, and reproducibility of the ICP-OES method for monitoring metal-ion release in saliva after removal of orthodontic appliances.

### 2.6. Statistical Analysis

Statistical analyses were conducted in IBM SPSS Statistics version 26.0 (IBM Corp., Armonk, NY, USA). Normality of the data was assessed with the Shapiro–Wilk test. Changes in salivary concentrations of cobalt (Co), nickel (Ni), Chromium (Cr), and titanium (Ti) across the three repeated measurements (T1, T2, and T3) were analyzed with one-way repeated-measures analysis of variance (repeated-measures ANOVA). Sphericity was evaluated with Mauchly’s test, and when violated, the Greenhouse–Geisser correction was applied. Pairwise comparisons were conducted with Bonferroni adjustment. Effect sizes were reported as partial eta squared (ηp^2^). Statistical significance was set at *p* < 0.05.

## 3. Results

In this pilot longitudinal exploratory clinical biomonitoring study, we focused on detecting metallic ions in biological fluids following long-term exposure to orthodontic materials. Table 2 presents the mean concentrations of the metals analyzed at the three time points, which were generally low. These levels decreased over time following the removal of the orthodontic appliance, except for nickel, which increased at T3 (three months after removal). To better understand the data distribution and since some means had large standard deviations, we also compared the median values with their respective means. Specifically, the median chromium concentration at T2 was similar to the reported mean, indicating that variability was not driven by extreme outliers but rather by natural variation within the studied population. Therefore, despite the relatively large standard deviation, the central tendency of the data remained consistent and reflective of the sample.

As expected in a pilot longitudinal study with a limited sample size, these estimates should be interpreted as preliminary and useful primarily for informing the design and sample size calculations of future studies.

**Figure 2:**
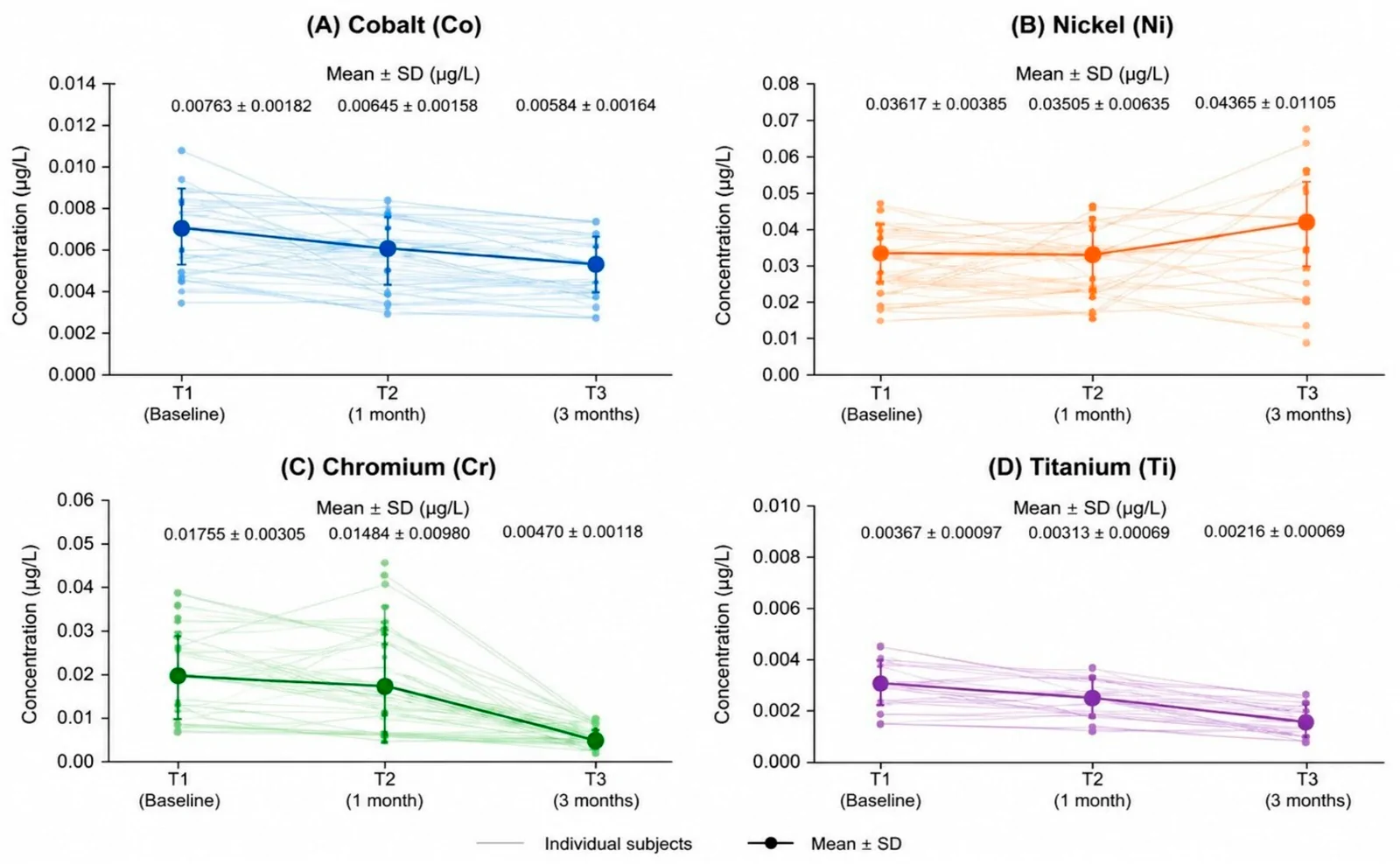
Temporal trends of metal ion concentrations in saliva after removal of fixed orthodontic appliances. Mean ± standard deviation (SD) for cobalt (**A**), nickel (**B**), chromium (**C**), and titanium (**D**) are shown at baseline (T1), 1 month (T2), and 3 months (T3). Thin lines represent individual subject trajectories.

Figure 2 illustrates the temporal evolution of salivary cobalt, chromium, nickel, and titanium concentrations during the three-month follow-up period after removal of fixed orthodontic appliances. In addition to the group mean ± standard deviation (SD), individual participant trajectories are shown to provide greater transparency regarding inter-individual variability. Overall, cobalt, chromium, and titanium concentrations declined over time, whereas nickel concentrations increased slightly at T3.

All variables met the assumption of normality according to the Shapiro–Wilk test (*p* ≥ 0.05). Mauchly’s test indicated that the sphericity assumption was met for cobalt (Co), nickel (Ni), and titanium (Ti) (all *p* ≥ 0.05), whereas it was violated for chromium (Cr) (*p* < 0.001). Therefore, the Greenhouse–Geisser correction was applied for chromium, while sphericity-assumed results were retained for the remaining analyses (Table 3).

Repeated-measures ANOVA revealed significant changes over time for cobalt (F(2,28) = 4.165, *p* = 0.026, ηp^2^ = 0.229), nickel (F(2,28) = 6.127, *p* = 0.006, ηp^2^ = 0.304), chromium (Greenhouse–Geisser-corrected: F(1.159,16.224) = 18.854, *p*< 0.001, ηp^2^ = 0.574), and titanium (F(2,28) = 13.691, *p* < 0.001, ηp^2^ = 0.494) (Table 4).

Bonferroni-adjusted pairwise comparisons showed a significant increase in nickel concentrations from T2 to T3 (*p* = 0.027). Chromium concentrations were significantly lower at T3 than at both T1 (*p* < 0.001) and T2 (*p* = 0.006). Similarly, titanium concentrations were significantly lower at T3 than at T1 (*p* < 0.001) and T2 (*p* = 0.008). No significant pairwise differences were observed for cobalt after Bonferroni adjustment (Table 5).

To facilitate interpretation of metal concentrations in saliva, the measured values were further expressed as an estimated theoretical oral ionic load. Concentrations (μg/mL) were converted using an average daily salivary volume of approximately 1500 mL, commonly reported for healthy adults. This calculation was intended solely to provide a descriptive estimate of the amount of metal ions potentially present in the oral environment under normal salivary flow conditions and to facilitate comparison across sampling times (Table 6).

It is important to emphasize that these estimates should not be interpreted as systemic intake, absorbed dose, body load, or toxicological exposure. Consistent with current human biomonitoring principles, salivary concentrations primarily reflect local conditions in the oral cavity and are influenced by salivary flow, oral clearance, biofilm interactions, and surface adsorption/desorption. Therefore, the values in Table 3 represent theoretical oral ionic loads, not measurements of systemic exposure. For context, Table 3 also includes reference values reported by the World Health Organization and other international organizations.

## 4. Discussion

Many previous studies have concluded that orthodontic appliances have corrosive potential in the oral cavity. Al-Horini et al. [16] note that mechanical and thermal changes in NiTi archwires can alter their surface properties and susceptibility to corrosion, potentially affecting ion release during and after treatment. Metal ions released by fixed appliances could act as allergens or cause serious adverse biological effects. Nickel and chromium are considered cytotoxic, mutagenic, and carcinogenic even at low concentrations [17,18].

The sliding of the archwire through the brackets causes friction between the archwire and the brackets, which can increase corrosion and release metal ions throughout orthodontic treatment. It is unknown whether the concentrations of these metal ions increase towards the end of treatment with fixed appliances.

Thus, the present study evaluated metal ion release at different time points at the end of orthodontic treatment by measuring ion concentrations in salivary samples. Matos de Souza et al. [10] stated that saliva is the most important sample for evaluating metal ion release because it is the first diluent in the human body and can be used for long-term chemical analysis.

Previous studies [6,18,19,20,21,22,23,24,25,26,27,28] have evaluated the release of metal ions at short intervals: before orthodontic appliance placement, and at hours, days, 1 to 4 weeks, and 1, 3, and 6 months after placement. However, a short period is not sufficient to evaluate the release of salivary metal ions from orthodontic appliances. This criticism of these studies is based on the findings from galvanic corrosion studies, which have concluded that corrosion increases with fatigue of the appliance, which, in an orthodontic appliance, is due to the continuous loading forces exerted on the appliance to move the teeth.

The results of this longitudinal pilot study indicate statistically significant differences in salivary concentrations of nickel, chromium, and titanium after removal of fixed orthodontic appliances and at 1 and 3 months thereafter. Notably, nickel levels increased at 3 months; however, this finding should be interpreted with caution. While delayed release associated with surface alterations generated during appliance removal is a plausible explanation, other factors could have contributed to this finding, including dietary and environmental nickel exposure, adsorption and desorption within the acquired salivary pellicle or oral biofilm, orthodontic material residues, and normal biological variability among participants. Given the absence of baseline measurements and a control group, the study design does not allow definitive attribution of this variation to orthodontic treatment.

Similarly, the progressive decline in chromium concentrations over time may be consistent with changes in the oral environment after appliance removal, including the cessation of mechanical and electrochemical interactions. These considerations should be considered tentative, given the study’s exploratory scope.

Importantly, salivary metal concentrations reflect local conditions in the oral cavity and cannot be directly extrapolated to systemic exposure. Consequently, no definitive conclusions about biocompatibility, safety, or potential toxicity should be drawn from these findings alone. Overall, the results should be considered preliminary and hypothesis-generating, underscoring the need for longitudinal studies using more robust methodologies.

The variations in nickel levels in the studies by Kocadereli et al. [19], Matos de Souza et al. [10] Arab et al. [8], Menezes et al. [28], Haugli et al. [29], Dwivedi et al. [30], Mikulewicz et al. [31], Urbutytė et al. [32] and Stoyanova-Ivanova et al. [33] could be explained by physiological and environmental factors, such as: time of day; pharmaceuticals; systemic health; nickel binding to epithelial cells, bacteria, and macromolecules in saliva; sampling method (stimulated vs. unstimulated saliva collection); the concentration of nickel and other metal ions in the air or soil, or in the food and water consumed; and the type of cooking utensils.

Most other studies [6,17,18,19,20,21,22,24,34,35] reported an increase in nickel ion concentrations after placement of orthodontic appliances.

Jurela et al. [24] found that salivary titanium levels significantly increased six months after the installation of orthodontic appliances, and the salivary levels of chromium and zinc decreased significantly after the installation of orthodontic appliances. These results are similar to those of our study: the release of ions is higher when device corrosion begins and decreases as the concentration of ions in the appliance declines due to corrosion or appliance removal.

Quadras et al. [36], Amini et al. [25], Nayak et al. [14], and Talic et al. [35] studied the release of metal ions in patients undergoing orthodontic treatment in a case–control study over a longer period, up to 18 to 32 months. Quadras et al. [36] observed that the release of chromium in saliva and blood serum fluctuated initially and then increased after 1.5 years. This is different from our study, which observed decreased Cr levels throughout this period. Comparison of mean chromium levels in saliva and blood serum between the study and control groups showed no differences before appliance insertion; however, after 1.5 years, a significant increase was observed, although the values remained within dietary limits and did not reach toxic levels. Similarly, Nayak et al. [14] reported a statistically significant increase of 17.92 ppb in salivary chromium ion concentration at the end of their study (10–12 months).

Talic et al. [35] found that Ni and Cr levels in the saliva of Saudi patients with orthodontic braces were lower than the toxic threshold and did not reach the dietary intake levels of these metals.

Furthermore, recent systematic reviews have consistently shown that fixed orthodontic appliances release metallic ions into saliva due to corrosion, friction, and physicochemical interactions in the oral environment. Urbutyte et al. [32] and the meta-analysis by Imani et al. [6] reported that salivary nickel and chromium concentrations generally increase during active orthodontic treatment. However, the detected levels remain below those associated with systemic toxicity. However, both reviews also highlighted considerable heterogeneity in sampling protocols, analytical techniques, and follow-up periods, underscoring the need for standardized longitudinal studies.

Unlike previous investigations that focused primarily on the active treatment phase, the present study evaluated salivary concentrations of Co, Ni, Cr, and Ti immediately after appliance removal and at the first and third months post-debonding. This post-treatment approach provides novel evidence on the persistence and clearance of residual metallic ions after appliance removal. The persistence of Ni at the third month may reflect residual corrosion processes, exposure of a freshly abraded metallic surface during debonding, or individual differences in salivary clearance.

The selection of saliva as the biological matrix is supported by recent systematic reviews that demonstrate its value as a non-invasive, reproducible, and clinically relevant biomonitoring fluid. Rodrigues et al. [37] emphasized that saliva enables repeated longitudinal assessment of biological changes throughout orthodontic treatment, while Bogdan-Andreescu et al. [38] highlighted its growing role in exposure assessment and metal biomonitoring. Likewise, Sallam et al. [39] concluded that salivary metal ion determination is a reliable approach for monitoring exposure to orthodontic alloys, despite methodological variability across published studies. Collectively, these findings support the methodological rationale of the present study and reinforce its contribution by characterizing salivary metal ion dynamics during the early post-treatment period. This stage remains largely underrepresented in the current literature.

Previous studies have shown that released metal ions can accumulate in various organs. The threshold concentrations that can cause toxicity in humans are unknown, so ion concentrations are typically compared with levels ingested through the daily diet [35,36,37,38,39,40]. In this longitudinal pilot study, the measured concentrations remained below the dietary exposure levels commonly reported by the World Health Organization. The daily doses are 2.4 μg/day for Co (actual intake often exceeds this figure, ranging between 5 and 40 μg/day), 300–600 μg/day for Ni, 50–200 μg/day for Cr, and 25–30 μg/day for Ti [1,31,34,41]. However, given the exploratory nature of the study and the limited sample size, these findings should be interpreted with caution and not regarded as definitive proof of long-term safety. Further longitudinal studies with appropriate statistical power may help to strengthen and confirm these findings.

This study has limitations that should be considered when interpreting the results. First, baseline measurements before orthodontic treatment were not obtained, which prevents intra-individual comparisons and a precise determination of whether the observed metal ion concentrations are directly associated with orthodontic therapy or reflect pre-existing physiological conditions. Second, the absence of a control group of untreated individuals limits the ability to establish reference values under equivalent conditions. It is important to note that this study was designed to characterize metal-ion concentrations following exposure to fixed appliances under real-world clinical conditions. Because trace metal concentrations in saliva are affected by numerous endogenous and exogenous variables, comparisons with untreated individuals should be interpreted with caution. Similarly, the small sample size (n = 15) is inherent to the design of an exploratory pilot study, whose main objective is to assess feasibility and estimate measurement variability. We acknowledge that this reduces statistical power and limits the generalizability of the findings. Regarding the statistical approach, appropriate methods for small samples and repeated measurements were applied, and the description was strengthened to improve methodological quality.

Consequently, the results should be interpreted with caution and considered exploratory. This pilot study, however, provides relevant preliminary information and lays the groundwork for future research using more robust longitudinal designs, larger sample sizes, pre-treatment assessments, and adequate control groups to strengthen the validity of the results.

Despite this limitation, the current study provides clinically relevant preliminary evidence of metallic ions in saliva after removal of fixed orthodontic appliances. Importantly, the post-treatment phase remains largely understudied in the existing literature, as most previous studies have focused on ion release during active orthodontic treatment or immediately after appliance placement. Therefore, assessing metallic ion levels at the end of treatment and following appliance removal provides new insights into whether these elements persist or are cleared from the oral environment.

Other potential limitations of this study include the lack of dietary control before saliva sampling, as the goal was to assess metal ion levels under real-world clinical conditions at the end of orthodontic treatment rather than in artificially restricted exposure scenarios. Trace metals such as cobalt, nickel, chromium, and titanium are common in the environment and often found in food and drinking water; therefore, short-term dietary restrictions would not reliably eliminate background exposure or accurately reflect the patient’s usual exposure profile. Additionally, dietary intake typically results in minimal, short-term changes in salivary concentrations compared to the ongoing contribution from the prolonged intraoral presence of orthodontic alloys. For this reason, our results were interpreted using reference values published by international health organizations, including the World Health Organization, which provide widely accepted benchmarks for assessing trace metal exposure in biological fluids under typical environmental conditions. This method helps contextualize the findings within a realistic exposure environment while maintaining the ecological validity of the biomonitoring assessment conducted at the end of orthodontic treatment.

Furthermore, another uncontrolled variable was the use of mouthwashes and fluoride exposure during the process. While these factors can influence the electrochemical behavior of orthodontic alloys, this study aimed to evaluate metal ion levels under real-world clinical conditions at the end of orthodontic treatment. Standardizing or restricting these variables could have decreased the study’s ecological validity and limited the applicability of the results.

In summary, this study analyzed changes in metal ion levels at the end of orthodontic treatment and immediately after appliance removal, focusing on a clinically relevant stage that has received limited attention in prior research.

## 5. Conclusions

In this pilot longitudinal clinical study, salivary levels of cobalt, chromium, and titanium decreased after removal of fixed orthodontic appliances, suggesting reduced metal-ion release during the post-treatment period. Nickel levels showed a slight increase at T3; however, this finding should be interpreted cautiously given the study’s exploratory nature. Although the measured concentrations remained below reported average dietary intake values, salivary measurements alone do not permit definitive conclusions about systemic exposure, safety, or toxicological implications.

These findings provide preliminary clinical observations of temporal changes in salivary metal-ion concentrations following orthodontic therapy. However, their interpretation is constrained by the pilot design and should be considered hypothesis-generating rather than confirmatory.

Further well-designed longitudinal studies with larger sample sizes, baseline measurements, and longer follow-up periods are needed to validate these preliminary findings and more accurately assess the clinical relevance of metal ion release among orthodontic patients.

## Figures and Tables

**Table 1:** Demographic and clinical characteristics of the study participants (n = 15).

Variable	Value
Participants	15
Age (years) mean ± SD	23.4 ± 5.4
Age range (years)	14–33
Female, n (%)	10 (66.7)
Male, n (%)	5 (33.3)
Orthodontic treatment duration (months), mean ± SD	25.7 ± 3.9
Treatment duration range (months)	18–30

Abbreviations: SD, standard deviation.

**Table 2:** Mean, median, and standard deviation of metal ion concentrations in saliva (μg/mL).

Metal Ion		T1	T2	T3
**Co**	Mean ± SD	0.00763 ± 0.00182	0.00645 ± 0.00158	0.00584 ± 0.00164
	Median	0.00692	0.00641	0.00616
**Ni**	Mean ± SD	0.03617 ± 0.00385	0.03505 ± 0.00635	0.04365 ± 0.01105
	Median	0.03643	0.03330	0.04784
**Cr**	Mean ± SD	0.01755 ± 0.00305	0.01484 ± 0.00980	0.00470 ± 0.00118
	Median	0.01773	0.01547	0.00430
**Ti**	Mean ± SD	0.00367 ± 0.00097	0.00313 ± 0.00069	0.00216 ± 0.00069
	Median	0.00369	0.00313	0.00228

**Table 3:** Assumption testing for repeated measures ANOVA.

Metal Ion	Mauchly’s W	χ^2^	df	*p*-Value	Greenhouse–Geisser ε	Sphericity Assumption	Statistical Approach
**Co**	0.933	0.904	2	0.636	0.937	Met	Sphericity assumed
**Ni**	0.676	5.097	2	0.078	0.755	Met	Sphericity assumed
**Cr**	0.274	16.821	2	<0.001	0.579	Violated	Greenhouse–Geisser correction applied
**Ti**	0.940	0.803	2	0.669	0.944	Met	Sphericity assumed

Abbreviations: df: degrees of freedom; ε: Greenhouse–Geisser epsilon.

**Table 4:** Repeated-measures ANOVA for changes in salivary metal ion concentrations over time.

Metal Ion	Degrees of Freedom	F	*p*-Value	Partial η^2^	Effect Size Interpretation
**Co**	2.28	4.165	0.026	0.229	Large
**Ni**	2.28	6.127	0.006	0.304	Large
Cr *	1.159, 16.224	18.854	<0.001	0.574	Large
**Ti**	2.28	13.691	<0.001	0.494	Large

* Greenhouse–Geisser-corrected degrees of freedom. Interpretation of partial η^2^: small = 0.01; medium 0.06; large ≥ 0.14.

**Table 5:** Bonferroni-adjusted pairwise comparisons between study time points.

Metal Ion	Comparison	Mean Difference	95%CI	Adjusted *p*-Value
**Co**	T1 vs. T2	0.00118	0.000 to 0.003	0.201
	T1 vs. T3	0.00179	0.000 to 0.004	0.072
	T2 vs. T3	0.00061	−0.001 to 0.002	0.938
**Ni**	T1 vs. T2	0.00112	−0.004 to 0.006	1.000
	T1 vs. T3	−0.00748	−0.016 to 0.001	0.101
	T2 vs. T3	−0.00860	−0.016 to −0.001	0.027 *
**Cr**	T1 vs. T2	0.00271	−0.004 to 0.010	0.932
	T1 vs. T3	0.01285	0.011 to 0.015	<0.001 *
	T2 vs. T3	0.01014	0.003 to 0.017	0.006 *
**Ti**	T1 vs. T2	0.00054	0.000 to 0.001	0.360
	T1 vs. T3	0.00151	0.001 to 0.002	<0.001 *
	T2 vs. T3	0.00097	0.000 to 0.002	0.008 *

* ANOVA *p* ≤ 0.05. Co = cobalt; Ni = nickel; Cr = chromium; Ti = titanium. T1 = appliance removed; T2 = one month after removal; T3 = three months after removal.

**Table 6:** Estimated theoretical oral ionic load based on salivary metal concentrations and the average daily salivary volume.

Metal Ion		T1	T2	T3	Reference Value *
**Co**	Concentration (μg/mL)	0.00763	0.00645	0.00584	-
	Estimated theoretical oral ionic load(μg/mL)	11.44	9.67	8.76	40–50
**Ni**	Concentration (μg/mL)	0.03617	0.03505	0.04365	-
	Estimated theoretical oral ionic load(μg/mL)	54.25	52.57	65.47	300–600
**Cr**	Concentration (μg/mL)	0.01755	0.01484	0.00470	-
	Estimated theoretical oral ionic load(μg/mL)	26.32	22.26	7.05	50–200
**Ti**	Concentration (μg/mL)	0.00367	0.00313	0.00216	-
	Estimated theoretical oral ionic load(μg/mL)	5.50	4.69	3.24	25–30

μg/mL = micrograms per milliliter; μg/day = micrograms per day. * Reference values represent dietary intake or exposure ranges reported by international health agencies and are provided solely for contextual comparison. They are not salivary reference values and should not be directly compared with estimates of systemic exposure.

## Data Availability

The original contributions presented in the study are included in the article; further inquiries can be directed to the corresponding authors.

## Abbreviations

The following abbreviations are used in this manuscript:

Co	Cobalt
Ni	Nickel
Cr	Chromium
Ti	Titanium
ICP-OES	Inductively Coupled Plasma Optical Emission Spectroscopy
WHO	World Health Organization
CIEAO	Research Center and Advanced Studies in Dentistry
HDPE	High-density polyethylene
ANOVA	Analysis of variance
